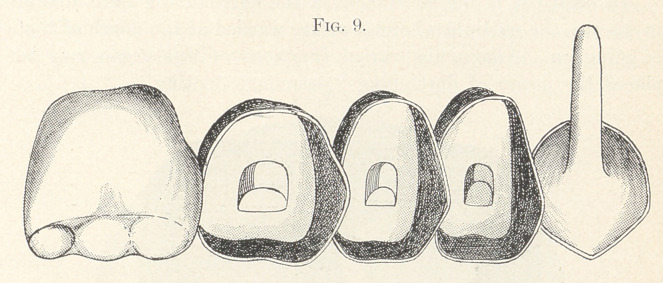# The Diatonic Tooth in Bridge-Work

**Published:** 1904-04

**Authors:** R. M. Sanger

**Affiliations:** East Orange, N. J.


### International Dental Journal.

Vol. XXV. April, 1904. No. 4.

### Original Communications.[1]

### THE DIATORIC TOOTH IN BRIDGE-WORK.[2]

BY R. M. SANGER, D.D.S., EAST ORANGE, N. J.

That crown- and bridge-work has come to stay no one will question, but that it has not reached perfection from either a hygienic or æsthetic stand-point is obvious to all of us. To the dentist who is worthy of that name a gold crown on any of the anterior teeth is a source of humiliation, and even the second bicuspid is covered with gold only when there is nothing more feasible. In fact, the bold display of gold crowns is rapidly becoming the sign manual of the dental parlor and the fakir, and I hope and believe that the time is not far distant when the self-respecting public, as well as the self-respecting professional man, recognizing the fact that a display of gold in the mouth is indicative of the parvenu and the shoddy, will cause a demand for some other and more æsthetic means of restoring their broken-down teeth to a condition not only of usefulness but of ornamentation. It is true that single crowns

[1] The editor and publishers are not responsible for the views of authors of papers published in this department, nor for any claim to novelty, or otherwise, that may be made by them. No papers will be received for this department that have appeared in any other journal published in the country.

[2] Read before the Academy of Stomatology, Philadelphia, November 24, 1903.

for the anterior teeth have reached a high state of perfection, and in the Logan, the Richmond, the Darby, and the half-collar crowns we have at our disposal methods of restoring those teeth which are almost ideal, but when perfectly sound anterior teeth are to be used as anchorage piers for bridges, the methods at our disposal for utilizing and at the same time preserving those teeth intact are somewhat faulty. The open-faced or window crowns so commonly used for this purpose are not only very unsightly, but in the majority of cases prove to be a delusion and a snare, their early failure usually being accompanied by a woful amount of destruction of tooth-substance, compelling the final loss of the natural crown and the construction of the pier on entirely different and more radical principles. The Carmichael and the Marshall systems offer a less conspicuous and possibly more effective method of retention, and if they prove to be all that they promise they will certainly be a welcome addition to our outfit, but their introduction is of so recent a date that we can only hope that time will prove them to be all that we desire.

Dr. F. L. Marshall, of Boston, describes his method, which he calls a staple crown, about as follows: With an enamel fissure bur cut grooves in the mesial and distal surfaces of the tooth just back of the contour, as deep as the fissure bur, then connect these grooves across the tooth by the use of an enamel bur, making this groove as deep as the other two. If the articulation is very close, grind off enough to allow for the thickness of the gold. Then knife-edge your tooth from the cross groove to the edge, as you would an artificial tooth before backing. Next select a piece of platino-iridium or 22-carat gold wire, a trifle smaller than the fissure bur, bend a right angle on the end long enough to fit one approximal groove, take the distance across the tooth between the grooves and bend another right angle in the wire, thus forming the staple (Fig. 1),

Fig. 1.

which gives the crown its name. Cut the ends of the staple the proper length to fit the grooves and allow the cross-section to be flush with the palatine surface of the tooth. A piece of pure gold is selected, a little larger than the crown, and one side of the staple is held against it, at right angles, with a pair of pliers, and it is caught with a little 22-carat solder. It is then placed in position on the tooth in the mouth and the gold burnished down to fit the lingual portion of the tooth and the approximal surfaces sufficiently anterior to the staple to allow of burnishing. Remove carefully and solder the staple to the crown around its entire length. Now replace the crown in the mouth and burnish carefully to place, trimming off all unnecessary gold. When a perfect adaptation is obtained, the piece is carefully removed and the outside is overflowed with solder to give rigidity; the entire free edge, however, to about the width of one thirty-second of an inch is kept free from solder to admit of burnishing when the final placing is done and before the cement is hard.

The Carmichael method, as I understand it, is about the same as the foregoing, except that the gold is burnished into the groove instead of using the staple.

In my own practice I have had frequent recourse to an old, but, because of its more heroic nature, less commonly used method than either of the above, with much satisfaction both to my patient and myself,—that is, the immediate anæsthetizing and removal of the pulp and the swaging or burnishing of a piece of gold to the palatine surface of the tooth, passing a platino-iridium pin through it and soldering, giving a pier as shown in Fig. 2, *a* and *b*. This

Fig. 2.

admits of the covering of the minimum amount of tooth-substance with gold and at the same time gives the maximum amount of strength to the pier. If an adjustable bridge is to be made, a platinum tube is used in place of the pin, and the pin, which is attached to the bridge, telescopes into the tube. In the earlier days the necessity for the administration of a general anæsthetic (gas in my practice) for the immediate removal of the pulp painlessly, was ofttimes a serious objection to the patient and to some operators, but with the introduction of cocaine and adrenalin chloride, the painless removal of the pulp by pressure anæsthesia has become such an easy and certain operation that I do not hesitate to do it whenever such an operation is indicated, believing that the subsequent results fully warrant a procedure which at first glance may seem very radical.

Next to the unsightly piers of your bridges are the solid gold cusps of the bicuspid and molar dummies, and it was to a method of overcoming this difficulty that I wished especially to call your attention to-night. By the use of the diatoric tooth instead of porcelain facings you can construct a bridge which is more æsthetic in appearance, simpler in construction, has greater strength, and is more readily repaired in case of fracture.

Using a bicuspid diatoric tooth for purposes of illustration, the procedure is as follows: The teeth are carefully selected to fit the case with as little grinding as may be necessary. The form in which they are made, with a long curve on the inner surface (Fig. 3, *a* and *b*), permits the cervico-lingual surface to fit the curve of

Fig. 3.

the average ridge with little or no grinding, but if any grinding is necessary, it should be done on the base of the tooth rather than on the morsal surface. A piece of pure gold plate about 32 gauge is cut to a size sufficient to cover the base of the tooth and project over the sides about one-eighth of an inch. This is laid on the base of the tooth and burnished to fit as nearly as possible, the edges being turned up all around to form a cup-like shape. A metal ring which will fit in a crown swager of the style shown in Fig. 4 is filled with hot modelling composition and the morsal surface of the diatoric tooth is pressed into it to a sufficient depth to hold it firmly (Fig. 5), and the whole plunged into cold water to harden the composition. (I procured my metal rings by cutting a small bicycle pump in sections). With the piece of gold in position on the embedded tooth, they are placed in the swager and covered

Fig. 4.

with cornmeal or some equally yielding substance, and swaged down until the gold cup fits the tooth accurately. Upon removal the gold is trimmed to the desired height around the edges, always allowing it to extend well up to the little holes on the approximal sides of the tooth, and then with a ball burnisher it is burnished into the central depression in the base of the tooth. The gold will be perforated when burnishing it into this hole, but the burnishing should be continued until the metal accurately fits the margins. It is then filled about one-third full with gold-foil, or a coil of pure

Fig. 5.

gold plate is placed in the cavity and burnished to fit. The gold cup is then removed from the tooth and the balance of the hole is filled with 20-carat solder until it is flush. This gives you a gold cup and pin which closely fits the diatoric tooth, holding it so firmly that it

Fig. 6.

Fig. 7.

Fig. 8

would almost keep its place without cementing. Fig 6 gives a side view of the cup and pin, Fig. 7 gives a front view of the same, and Fig. 8 shows the tooth in position in the gold cup. With the teeth in the cups, but not fastened, they are assembled on the cast and waxed to each other and to the piers on the lingual side. The assembled piece is then carefully placed in the mouth, and any error in the occlusion is corrected by allowing the patient to bite the teeth to place. The diatoric teeth are then removed from the cups, and the piers and cups are taken from the mouth in an impression of terra-plastica or some suitable investing material. This gives you the pieces invested and ready to solder at the approximal surfaces. For additional strength a piece of gold plate is laid across the lingual aspect from pier to pier and the whole overflowed with solder. When this is done the work is allowed to cool slightly, and

Fig. 9.

it is then plunged into cold water, which disintegrates the investing material and allows the work to come away clean and without injury to the fine edges. The polishing should be done with the porcelain teeth in position to prevent possible injury to the fine edges of the gold cups, but they should not be fastened permanently in the cups until everything is completed and ready for the mouth, thus avoiding dirty joints. (Fig. 9 shows the work ready for the final cementing of the diatoric dummies.) The teeth may be permanently fastened in the cups with oxyphosphate of zinc, gutta-percha, after the method devised by Dr. George Evans, and which I have found very satisfactory, or by the use of powdered sulphur, after the manner of attaching English tube-teeth to gold plates. In case of the fracture of one of these dummies, the repair can be quickly and easily done in the mouth, as the diatoric teeth are readily duplicated, but the danger of fracture is very remote, as the porcelain is at no time subjected to heat, and you have the full thickness of the tooth incased in gold to withstand the force of mastication. As I intimated at the beginning of this description, this method gives you a maximum degree of strength, a minimum display of gold, and an occlusion which is well-nigh perfect, and I am sure that the æsthetic appearance of a bridge constructed in this way will appeal to you all.

## Figures and Tables

**Fig. 1. f1:**
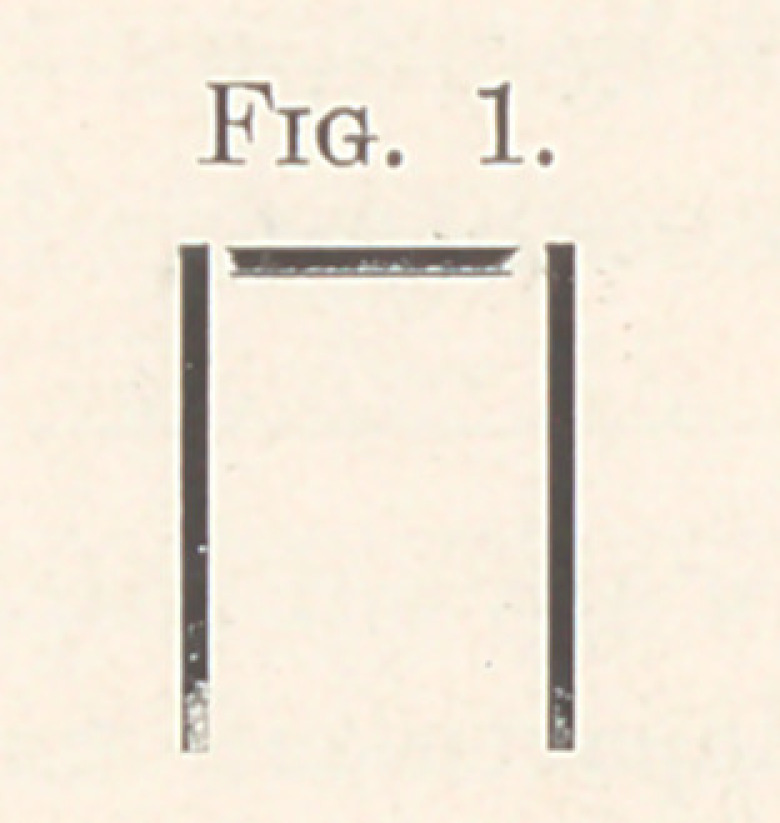


**Fig. 2. f2:**
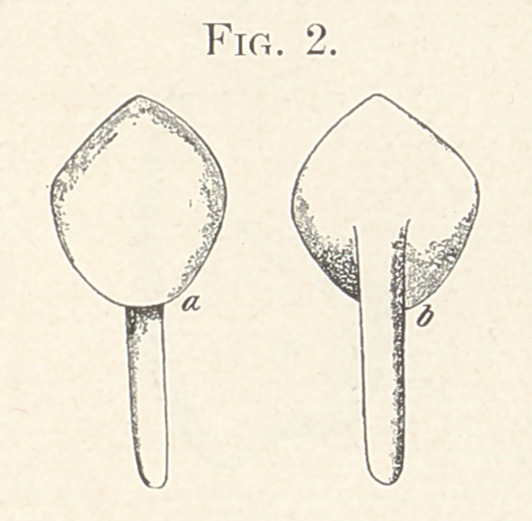


**Fig. 3. f3:**
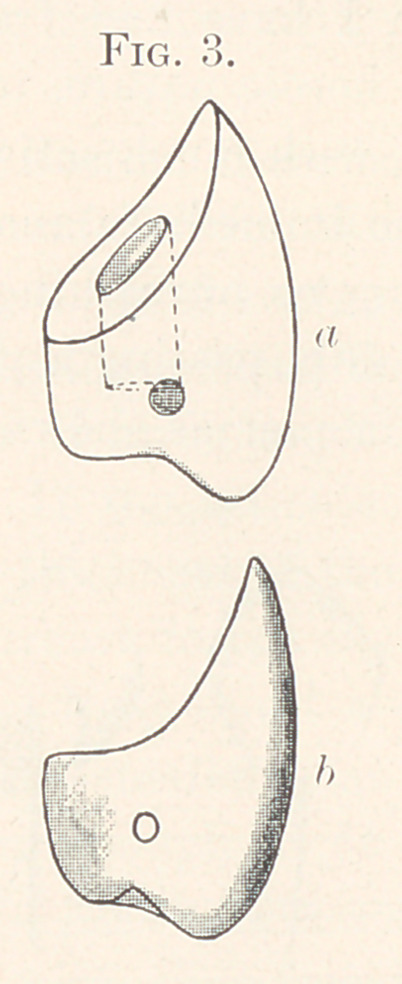


**Fig. 4. f4:**
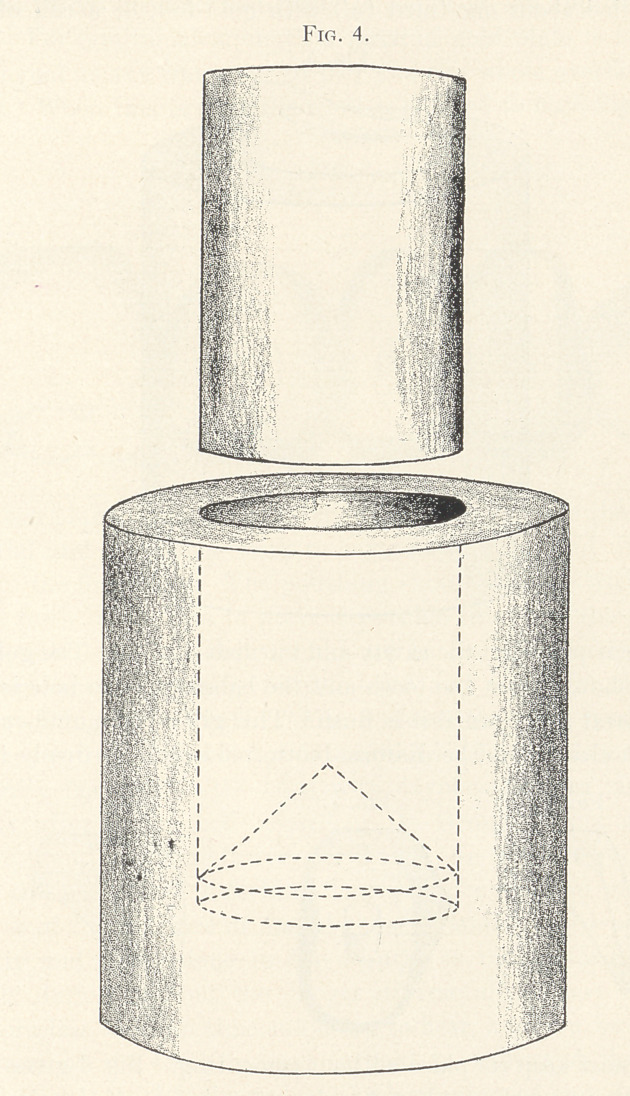


**Fig. 5. f5:**
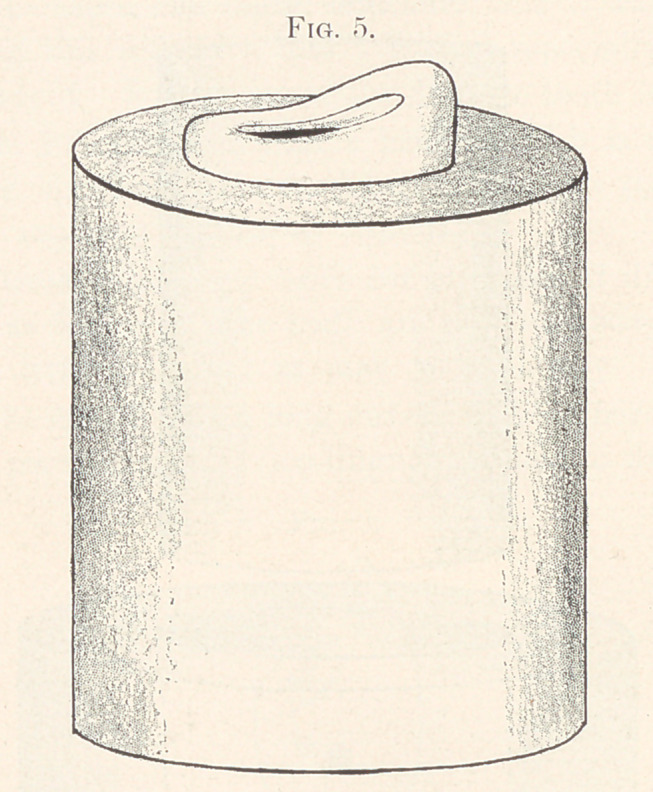


**Fig. 6. f6:**
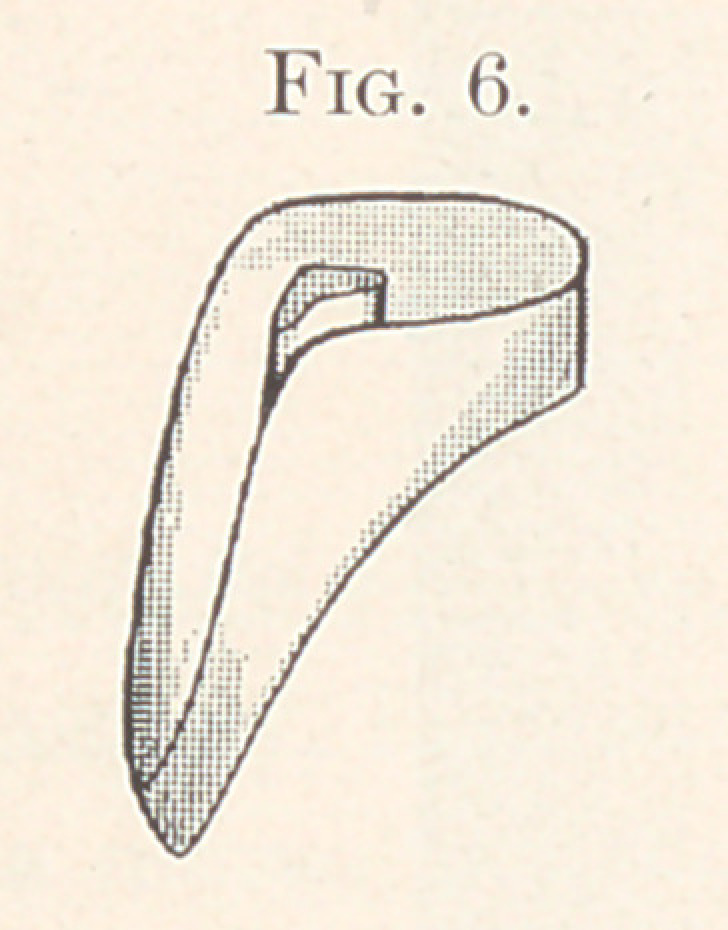


**Fig. 7. f7:**
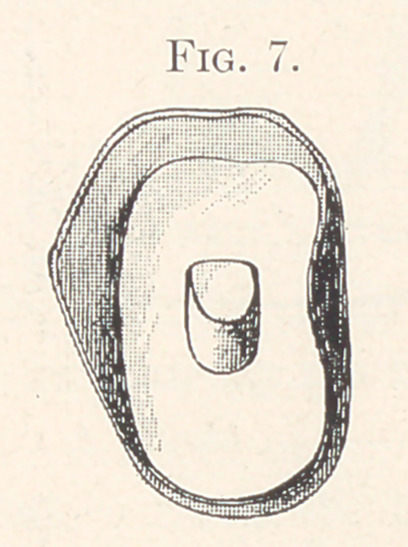


**Fig. 8 f8:**
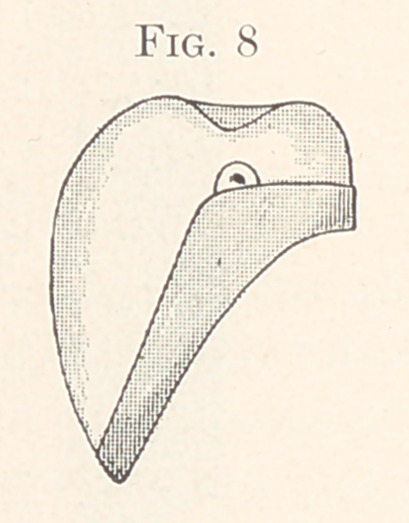


**Fig. 9. f9:**